# Metal Oxide Nanocomposites as Next-Generation Antimicrobial Agents Against Oral Cariogenic Pathogens: Mechanistic Actions of Ag–ZnO and Cu–ZnO on *S. mutans* and *S. sobrinus*

**DOI:** 10.3390/ma19081634

**Published:** 2026-04-19

**Authors:** Mohamed I. Ahmed, Anna Nowak, Mateusz Dulski, Aleksandra Strach, Aleksandra Zielińska, Monika Paul-Samojedny, Izabela Potocka, Krzysztof Matus, Daniel Wasilkowski

**Affiliations:** 1 Institute of Biology, Biotechnology, and Environmental Protection, Faculty of Natural Sciences, University of Silesia in Katowice, 40-032 Katowice, Poland; mohamed.ahmed@us.edu.pl (M.I.A.); anna.m.nowak@us.edu.pl (A.N.); izabela.potocka@us.edu.pl (I.P.); 2 Institute of Material Engineering, Faculty of Science and Technology, University of Silesia in Katowice, 41-500 Chorzów, Poland; mateusz.dulski@us.edu.pl; 3 Doctoral School, University of Silesia, Bankowa 14, 40-032 Katowice, Poland; aleksandra.strach@us.edu.pl; 4 Faculty of Pharmaceutical Sciences, Medical University of Silesia, 41-200 Sosnowiec, Poland; azielinska@sum.edu.pl (A.Z.); mpaul@sum.edu.pl (M.P.-S.); 5 Materials Research Laboratory, Silesian University of Technology, 44-100 Gliwice, Poland; krzysztof.matus@polsl.pl

**Keywords:** oral pathogenic bacteria, nanocomposites, MONC–bacterial cell interactions, ATR-FTIR spectroscopy, TEM/SEM imaging

## Abstract

**Highlights:**

- Stable, phase-pure MONCs with Ag^+^/Cu^2+^ doped on the ZnO NPs were synthesized.
- Acquired nanocomposites exhibited antimicrobial activity against *Streptococcus* spp.
- Synthesized MONCs interacted with specific bacterial cell functional groups.
- MONCs disrupted the structure and function of the outer layers of treated bacteria.
- Antibacterial effects of MONCs depended mostly on metal ion release.

**Abstract:**

Oral infections caused by antibiotic-resistant bacteria represent an emerging biomedical hazard and growing challenge for modern dentistry. To address this issue, Ag– and Cu–ZnO nanocomposites (NCs) were synthesized using ZnO carrier to combat the oral pathogens *Streptococcus mutans* and *Streptococcus sobrinus*. A comprehensive analysis of chemically synthesized metal oxide nanocomposites (MONCs) was performed, combining physicochemical characterization (TEM, XRD, ζ-potential, DLS, pH, and PFO/PSO kinetic models) with biological toxicity assessment (MIC, ATR–FTIR, SEM, and FAMEs) to better understand their antimicrobial mechanisms. The results confirmed that the synthesized nanoproducts fulfill the criteria for nanomaterials (NMs) (particle size < 100 nm). Among them, Ag–ZnO exhibited the highest antibacterial activity against both strains (MIC = 50 mg L^−1^). Kinetic modeling revealed faster and more efficient Ag ion release from Ag–ZnO NCs compared to Cu from Cu–ZnO NCs. Molecular analyses indicated strong MONC–bacterial interactions at the cell surface, leading to changes in protein secondary structures, alterations in lipid composition, and disruption of Gram-positive bacterial membranes. Additionally, Ag–ZnO inhibited chain and cluster formation in both bacterial species, while Cu–ZnO affected only *S. sobrinus*. Overall, Ag– and Cu–ZnO NCs show strong potential as antimicrobial agents against oral pathogens.

## 1. Introduction

Modern dental implantology is facing a growing problem of peri-implant infections, which are one of the leading causes of implant failure. Peri-implantitis, which develops because of the colonization of implant surfaces by pathogenic biofilms, leads to chronic inflammation, bone resorption, and eventual implant loss [1,2,3]. This process is particularly difficult to control because the microorganisms that form the biofilm exhibit increased tolerance to traditional antimicrobial agents. The scale of the problem is significant: according to data from the World Health Organization, oral diseases affect over 3.5 billion people worldwide, and in 2022, oral cancers caused nearly 200,000 deaths. Pathogenic biofilms develop through the adhesion of microorganisms to tooth surfaces and biomaterials, with bacteria from the genera *Streptococcus*, *Veillonella*, *Actinomyces*, and *Neisseria* dominating among the early colonizers [3,4].

The situation is further complicated by the global rise in antimicrobial resistance (AMR). Oral pathogens are increasingly exhibiting multidrug resistance, which limits the effectiveness of standard therapies and complicates infection control, especially in clinical settings [5,6,7,8,9]. Since the mid-20th century, the rate of bacterial resistance acquisition has outpaced the rate of new drug development, making AMR one of the most serious challenges in modern pharmacotherapy. According to CDC data, in 2019 alone, AMR contributed to nearly 5 million deaths worldwide. In response to this challenge, the WHO updated its list of priority bacterial pathogens in 2024, including, among others, methicillin-resistant *Staphylococcus aureus* (MRSA), β-lactamase-producing *Enterobacterales*, carbapenem-resistant *Acinetobacter baumannii*, fluoroquinolone-resistant *Salmonella typhi*, and vancomycin-resistant enterococci (VRE) [10,11,12,13].

In this context, nanomaterials, whose unique physicochemical properties enable effective interaction with bacterial cells, are of particular interest [14,15,16]. The antimicrobial activity of nanoparticles is primarily due to the controlled release of metal ions and their direct interactions with the cell surface [17,18]. The first point of contact is the cell wall, a key protective structure of bacteria. Interactions with its components lead to the formation of pores in the membrane, destabilization of cell integrity, and translocation of ions into the interior, initiating a cascade of processes including the generation of reactive oxygen species, metabolic disruption, DNA and ATP damage, and growth inhibition [17,18,19,20]. Notably, metallic NPs and metal oxide NMs are known for their potential for generating highly reactive oxygen species (ROS): singlet oxygen (^1^O_2_), superoxide anion (O_2_·^−^), hydroxyl radical (·OH) and ion (OH^−^), and hydrogen peroxide (H_2_O_2_). This process induces oxidative stress in microbial cells through catalytic oxidation, resulting in lipid peroxidation, membrane destabilization, protein oxidation, or DNA/RNA molecule damage, disrupting cell functioning and viability [21,22]. Different NPs and NMs have been shown to stimulate the production of ROS, such as Ag NPs [23], TiO_2_ NPs [24], and CuO NPs [25] or Fe-ZnO NMs [26]. More detailed analyses were presented for micro-layered calcium hydroxide NPs [27] and Co-doped ZnO quantum dots [28] to generate O_2_·^−^, ^1^O_2_ and ·OH. This allows nanomaterials to bypass classical resistance mechanisms, inhibit biofilm formation, and act synergistically with antibiotics [14,15,16,29].

Previous studies have primarily focused on single metal nanoparticles (Ag, Au, Zn, Cu, Ti, Si) and metal oxides (e.g., ZnO, CuO, TiO_2_, SnO_2_, Fe_3_O_4_, Mn_3_O_4_), which exhibit significant antimicrobial activity [29,30,31]. For instance, Ag nanoparticles demonstrated antimicrobial effectiveness against *Escherichia coli*, *Pseudomonas fluorescens*, *Saccharomyces cerevisiae* [32], and showed higher activity against Gram-negative bacteria (*E. coli*, *Klebsiella pneumoniae*, *Pseudomonas aeruginosa*) than Gram-positive strains (*Staphylococcus aureus*, *Bacillus subtilis*) [33,34]. The antimicrobial potential of Cu NPs has been reported against *E. coli* [35] as well as *E. coli*, *Salmonella enteritidis*, *Staphylococcus epidermidis*, *S. aureus*, and *P. aeruginosa* [36,37]. Au NPs are also effective against a broad range of bacteria, including *Corynebacterium pseudotuberculosis*, *S. aureus*, *S. epidermidis*, *B. cereus*, *E. coli*, *K. pneumoniae,* and *P. aeruginosa* [38,39,40]. Metal oxide nanoparticles further demonstrate strong antimicrobial activity. TiO_2_ NPs inhibited the growth of *E. coli* and *S. aureus* [41] and were effective against *S. epidermidis*, *B. subtilis*, and *E. coli* [42], while ZnO NPs showed activity against *S. aureus*, *S. epidermidis*, *E. coli*, and *P. aeruginosa* [43,44,45]. Promising antimicrobial effects were also reported for CuO and Cu_2_O NPs against multidrug-resistant *Acinetobacter baumannii*, *S. aureus*, *E. coli*, and oral pathogens [46,47,48,49], as well as for SnO_2_ NPs against *E. coli* and *B. subtilis* [50]. However, nanocomposite systems integrating two or more materials to enhance functional properties are attracting increasing attention, as synergistic effects often lead to significantly improved biological activity [51,52,53,54]. This strategy is particularly promising for protecting implant surfaces against bacterial colonization. For example, Žalnėravičius et al. [52] reported a Fe_3_O_4_–Au nanocomposite stabilized with D, L-methionine that yielded ultra-small NPs (1.8 nm) with high antibacterial activity against Gram-negative (*Acinetobacter baumannii*, *S. enterica*) and Gram-positive bacteria (MRSA *Staphylococcus aureus*, *Micrococcus luteus*). Similarly, Fe–Cu oxide nanocomposites exhibited enhanced antibacterial activity compared to single oxides [53], while LDPE modified with Ag–Cu–ZnO–Fe_2_O_3_ NPs achieved 99% antimicrobial effectiveness against pathogenic bacteria and fungi [54]. Polymer-stabilized metal NPs also demonstrated improved antimicrobial performance. Ag NPs stabilized with polyvinylpyrrolidone (PVP) were effective against both Gram-negative and Gram-positive bacteria, although increased MIC values were observed for selected strains [55,56]. Additionally, Ag NPs stabilized with BPEI, citrate, or PVP inhibited β-galactosidase activity in *E. coli* more effectively than free Ag^+^ [57], and phenylboronic acid-modified Au NPs showed broad-spectrum activity against multidrug-resistant bacteria [58]. Synergistic antimicrobial effects were reported for Ag NPs combined with selected antibiotics and for β-lactam antibiotics conjugated with Au NPs, whereas no enhancement was observed for Fe_3_O_4_ NPs combined with tobramycin [55,59,60,61].

Nowadays, nanotechnology plays an essential role in the development of advanced nano(bio)materials for biomedical and pharmaceutical applications. Despite their promising therapeutic potential, the unique physicochemical properties of NMs may significantly influence their interactions with biological systems, raising important concerns regarding safety and biocompatibility [62,63]. Nanomaterials have been shown to exert varying effects on cell viability, cytotoxicity and genotoxicity, depending on their composition, concentration and the type of target cells. Ag NPs, for example, demonstrated cytotoxic effects on B16-F10 (mouse melanoma cell line), MCF-7 (human breast cancer cell line), HEPG2 (human liver cancer cell line human liver cancer cell line) and HeLa cancer cells (human cervical cancer cell line), while normal CHO-K1 (Chinese hamster ovarian cells) cell line was less affected [64]. Similarly, Ag and ZnO NPs reduced cell viability, whereas TiO_2_ and CeO_2_ NPs showed minimal cytotoxicity [65]. Other studies indicated low cytotoxic effects of ZrO_2_ NPs on normal V-79 (lung normal cells from Chinese hamster) cells [66], while Au nanorods and graphene quantum dots decreased viability in multiple cell lines, with normal cells often being more sensitive [67,68]. Nanoparticles can also induce genotoxic effects, including DNA damage and chromosomal aberrations, as observed for Ag, ZnO NPs, and Au nanorods in various cancer and normal cells [64,65,68,69].

The present study aimed to engineer and evaluate the antimicrobial efficacy of novel metal oxide nanocomposites (MONCs), specifically, Ag_3_O_4_-ZnO (Ag–ZnO) and Cu_2_O-ZnO (Cu–ZnO). While previous work has primarily focused on metal nanoparticles or metal oxides individually, the relationships between ion immobilization, nanocomposite structure, ion release kinetics, and bactericidal efficacy remain largely unexplored. By precisely incorporating Ag^+^ and Cu^2+^ ions into a ZnO matrix, we investigated how the composition and surface properties of the nanocomposites influence antimicrobial activity against the clinically relevant oral pathogens *Streptococcus mutans* (ATCC 25175) and *Streptococcus sobrinus* (ATCC 33478). A comprehensive physicochemical characterization included assessment of the MONCs’ shape and size by transmission electron microscopy (TEM), crystallographic structure by X-ray diffraction (XRD), zeta (ζ) potential, hydrodynamic diameter by dynamic light scattering (DLS), pH, and ion release kinetics in a microbial environment. Antimicrobial activity was quantified by MIC determination, and nanocomposite-bacterial cell interactions were investigated using ATR-FTIR spectroscopy, FAME profiling, and surface morphology visualization (SEM). This approach allows for a comprehensive evaluation of the associations between the physicochemical properties of the nanocomposites and their biological effects.

## 2. Materials and Methods

**Figure 1:**
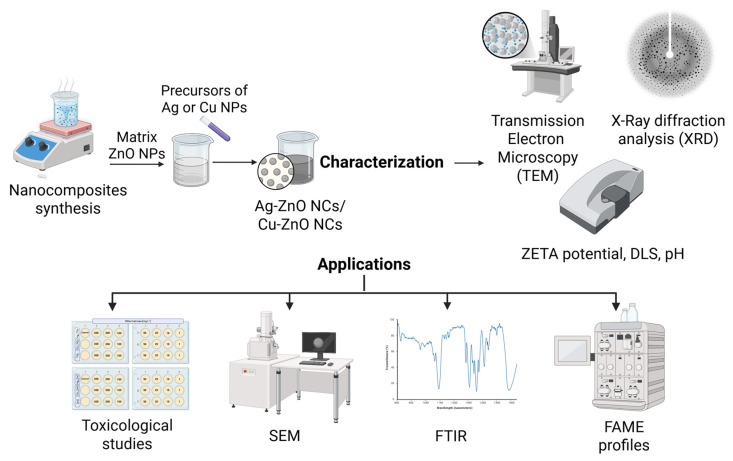
General schematic workflow of the integrated multidisciplinary methodology employed in this study. NPs: nanoparticles; NCs: nanocomposite; DLS: Dynamic Light Scattering; SEM: Scanning Electron Microscope; FTIR: Fourier Transform Infrared Spectroscopy; FAME: Fatty Acid Methyl Esters. (Created in BioRender. Wasilkowski, D. (2026) https://BioRender.com/52zvgrh, accessed on 14 April 2026).

The methodology proposed in this work adopts a multifaceted, integrated approach (Figure 1), including synthesis and physicochemical characterization of nanocomposites as well as microbial-nanoproduct interactions, including biomolecules, within a multidisciplinary workflow.

### 2.1. Synthesis of Metal Oxide Nanocomposites

The silver/zinc oxide and copper oxide/zinc oxide nanocomposites (Ag– and Cu–ZnO NCs) were synthesized through a chemical reduction in an aqueous solution (controlled conditions: air, temperature 100 °C, stirring 130 rpm) according to Nowak et al. [70]. In the first step, an aqueous solution of the alkaline reducer 1% NaOH (purity > 99.99%; Avantor Company, Gliwice, Poland) was added to a 1% solution of a commercially available nano-zinc oxide powder (ZnO NPs, <50 nm; Sigma-Aldrich Merck, St. Louis, MO, USA) serving as the matrix. To the metal oxide matrix, the 1% stock solution of AgNO_3_ and (purity > 99.9999% with trace metal basis; Merck Life Science Co., Darmstadt, Germany) and Cu(CH_3_COO)_2_·H_2_O (assay 98.0–102.0%; VWR International Co., Gdańsk, Poland) was added dropwise, using the respective ion precursors. Additionally, the synthesis of Cu–NPs required the use of 2% L-ascorbic acid (Biomus sp z.o.o., Lublin, Poland) as the reductant for copper ions. Finally, the resulting solution of metal-oxide nanocomposite (MONCs) was mixed on a magnetic stirrer at 100 °C for 2 h, and the NC product was obtained by filtration through a polyethylene membrane, and subsequently dried in air at room temperature.

For each experiment, the stock solution of MONCs was prepared with a concentration of 500 mg L^−1^, followed by sonication (20 kHz, 5 min), before dilution for each use.

### 2.2. Characterization of Engineered MONCs

#### 2.2.1. TEM Imaging with Energy-Dispersive X-Ray Spectroscopy

TEM microscopy was used to examine the morphology of the newly synthesized MONCs. For this purpose, the NC powder was suspended in ethanol, and the solution was ultrasonicated using a homogenizer (Omni Sonic Ruptor 400; PerkinElmer, Kennesaw, GA, USA) to disperse agglomerates. The resulting MONC suspension was deposited onto an amorphous carbon film supported on a copper grid (300 mesh). Particle size and morphology were characterized by high-resolution transmission electron microscopy (HR-TEM) and scanning transmission electron microscopy (STEM) with high-angle annular dark-field (HAADF) imaging. The chemical composition of the samples was determined using energy-dispersive X-ray spectroscopy (EDS). All measurements were performed on a Titan 80-300 FEI microscope equipped with an EDAX EDS detector (FEI Company, Hillsboro, OR, USA) at an acceleration voltage of 300 kV.

#### 2.2.2. X-Ray Diffraction Analysis

X-ray diffraction (XRD) of the synthesized MONCs, as well as the matrix (ZnO NPs), as a powder, was performed to determine their crystal structure and phase composition. The Empyrean PANalytical X-ray diffractometer (Malvern, Almelo, The Netherlands) was used. The powder patterns were recorded in the Bragg–Brentano geometry using CuKα radiation (λ = 1.54 Å). The analysis was carried out in the HighScore software (version 4.1) using data from the Crystallography Open Database (COD). The crystallite size was estimated using the Scherrer Equation (1):

*D* = k*λ*/*β* cos *θ* (1)

where *D* is the crystallite size

k = 0.94 is the shape factor, corresponding to spherical particles
*λ* is the X-ray radiation wavelength
*β* is the width of the peak at half of its maximum intensity
*θ* is the diffraction angle.

#### 2.2.3. ζ-Potential, DLS, and pH of MONC/NP Solutions

Surface charge (ζ-potential) and hydrodynamic diameter of MONCs and ZnO NPs were determined by dynamic light scattering (DLS) using a Litesizer 500 (Anton Paar GmbH, Graz, Austria) at the concentration providing maximum stability, following the instrument guidelines. The pH of the aqueous suspensions was measured at room temperature (20–22 °C) using pH-Fix test strips (Ref. 92110) according to the manufacturer’s instructions (Macherey-Nagel GmbH & Co. KG, Dueren, Germany).

### 2.3. Microbial Strains and Culture Conditions

The model microorganisms chosen in this study were obtained from the American Type Culture Collection (ATCC). The basic characteristics and selective culture media are shown in Table 1.

Prior to use, these isolates were thawed from −80 °C by placing them at room temperature (20–22 °C), and then directly transferred to the selective broth media BD BACTO^™^ Brain Heart Infusion (BD 237500), followed by incubation at 37 °C and 130 rpm for 24 h according to the product sheet. In the microbial studies, for each experiment, cells corresponding to the half-time (t_1_/_2_) of the mid-exponential growth phase (log phase) were taken. This approach is justified, as it ensures the use of rapidly growing cells with a species-specific generation time, while maintaining consistent cell size and protein content.

### 2.4. Toxicological Studies

#### 2.4.1. Evaluation of the Potential Toxicity of MONCs Against Microbial Cells

**Figure 2:**
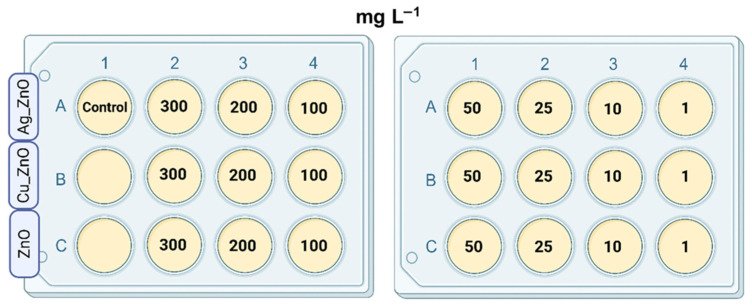
Scheme of 12-well plates for the MONC/matrix treatment experiment at NCs/NPs concentrations ranging from 1 to 300 mg L^−1^ and in the control. (Created in BioRender. Wasilkowski, D. (2026) https://BioRender.com/307m2v1, accessed on 14 April 2026).

The potential toxicological effect of engineered MONCs against the studied bacterial species was determined by the minimum inhibitory concentration (MIC). Additionally, the effect of matrix ZnO NPs on microbial cells was assessed. Before treating microbial cells, serial concentrations of MONCs/NPs in the tested range (300, 200, 100, 50, 25, 10, and 1 mg L^−1^) were prepared. For each bacterial strain and MONCs and ZnO NPs concentration, BD 237500 broth medium was dispensed into the wells of 12-well plates. Subsequently, an appropriate volume of the working solution of MONCS or ZnO NPs, prepared in water, was added to each well. The 12-well plates were prepared to achieve a final volume of 3 mL per well. The control well contained only medium without MONCs/NPs. Next, the washed biomass (equal to the mid-exponential growth phase) of bacteria was added to each well until OD_600_ = 0.1 (~10^7^ CFU mL^−1^) was reached. The plates with the samples were prepared as shown in Figure 2 and then incubated at 37 °C with shaking at 110 rpm for 24 h of treatment. Subsequently, the MIC was defined as the lowest concentration (in mg L^−1^) of MONCs or NPs itself that inhibits the growth of a given microbial strain (compared to the control, no microbial growth was observed, the culture media remained clear, with no turbidity detected) [71,72,73]. The analyses were performed in three technical replicates.

#### 2.4.2. Active Ag^+^ and Cu^2+^ Ion Release Kinetics

The release rates of Ag^+^ and Cu^2+^ ions from Ag–ZnO and Cu–ZnO MONCs at MIC concentrations were evaluated in BD Difco Bacto Brain Heart Infusion medium (237500) for *Streptococcus* spp. with respect to MONCs exhibiting antibacterial properties (equal to MIC). Samples (10 mL) were incubated under bacterial cultivation conditions (37 °C, 110 rpm) and collected after 0.5–48 h. Following centrifugation (4700 rpm, 10 min), the supernatants were transferred for analysis. Ag^+^ and Cu^2+^ concentrations were determined according to Dulski et al. [74] using a Thermo Scientific iCE 3000 Series Atomic Absorption Spectrometer (Thermo Fisher Scientific GmbH, Bremen, Germany) equipped with an air–acetylene flame (flow rate 0.9 L min^−1^; torch height 7.0 mm for Ag^+^ and 6.2 mm for Cu^2+^). Measurements were performed at 328.1 nm (Ag^+^) and 324.8 nm (Cu^2+^) with a lamp current of 75%, spectral slit width of 0.5 nm, and aspiration and measurement times of 4.0 s. Prior to FL-AAS analysis, Ag^+^ and Cu^2+^ samples were diluted 8- and 10-fold, respectively, with ultrapure Millipore water. All measurements were conducted in triplicate using deuterium background correction. Ion concentrations were calculated from calibration curves with blank correction using Ag^+^ (0.313–2.5 mg L^−1^) and Cu^2+^ (0.3–3 mg L^−1^) standard solutions prepared from Certipur stock solutions (1000 mg L^−1^; Merck Life Science Sp. z o.o., Darmstadt, Germany). The amount of Ag^+^ or Cu^2+^ absorbed was determined according to Equation (2) [75]:

(2) $$q_{t}\left({mg\;g}^{-1}\right)=\frac{\left(C_{t}-C_{0}\right)\cdot V}{W}$$

where *q_t_* is the amount of adsorbate released into the environment at time *t* (mg g^−1^), W is mass of the adsorbent (g), *C*_0_ is the initial concentration of the adsorbate (mg L^−1^) and is equal to 0, *C_t_* is the concentration of the adsorbate at time *t* (mg L^−1^), and *V* is the volume of the solution (L).

Finally, the rate of adsorption in liquid-solid interactions was estimated with two widely used adsorption kinetic models [75,76]:

Pseudo-first-order (PFO) expression:

(3) $$q_{t}\left({mg\;g}^{-1}\right)=\;q_{e\;}\left(1-e^{-k_{1}t}\right)$$

Pseudo-second-order (PSO) expression:

(4) $$q_{t}\left({mg\;g}^{-1}\right)=\;\frac{q_{e}^{2}k_{2}t}{1+q_{e}k_{2}t}$$

where *q_t_* is the amount of ions released into the environment at time *t* (mg g^−1^), *q_e_* is the amount of ions at equilibrium (mg g^−1^), *k*_1_ is the rate constant for the PFO model (h^−1^), *k*_2_ is the rate constant for the PSO model (g mg^−1^ h^−1^), and *t* is the adsorption/desorption time (h).

These models were selected to distinguish adsorption kinetics governed primarily by diffusion (PFO) from those controlled by chemical interactions or surface complexation (PSO) [77,78].

#### 2.4.3. Bacterial-MONC Interaction (ATR-FTIR)

The sample preparation method for spectroscopic analysis in the first step enabled the cultivation of living cells treated with MONCs or untreated control cells for 4 h. Then, the samples were washed with ultrapure water (H_2_O_MP_), centrifuged (5000 rpm, 20 min), and the pellet was freeze-dried at −15 °C for 18 h using a lyophilizer (Alpha 2-4 LSCbasic, Martin Christ Gefriertrocknungsanlagen GmbH, Osterode am Harz, Germany) [79]. Lyophilized samples were analyzed using Fourier Transform Infrared (FTIR) spectroscopy with an Agilent Cary 640 FTIR spectrometer (Agilent Technologies, Inc. Headquarters, Santa Clara, CA, USA), equipped with a standard source and a DTGS Peltier-cooled detector. Spectra were collected with a GladiATR diamond accessory over the spectral range of 400–4000 cm^−1^. For each strain, ten spectra were acquired for both control and MONC-treated samples. Each spectrum was accumulated over 16 scans with a spectral resolution of 4 cm^−1^. Post-processing of the infrared data was conducted using Win-IR Pro (v2.96) software, which applied baseline correction, removed water and carbon dioxide interference, and performed ATR correction (refractive index = 1.388) [80]. Further spectral pre-processing included second-derivative transformation using a 13-point Savitzky–Golay algorithm [81], followed by vector normalization. The application of second-derivative processing enhances multivariate data analysis by reducing non-chemical interferences such as optical and scattering artifacts [82,83]. Further interpretations were restricted to the spectral ranges of 1000–1800 cm^−1^ for specific vibrational bands presented in Table 2. These quantitative analyses were visualized using box-and-whisker plots [84,85,86].

#### 2.4.4. Surface Morphology of Bacterial Cells (Scanning Electron Microscopy, SEM)

The surface morphology of bacterial cells, following exposure to MONCs and untreated control, was visualized using a Hitachi SU-8010 field emission scanning electron microscope (FE-SEM; Hitachi High-Tech Corporation, Tokyo, Japan). Sample preparation was performed according to the protocols described by Metryka et al. [88] and Fischer et al. [89] after 4 h of treatment in culture medium. After washing with sterile H_2_O_MP_, the pellet was fixed in 2.5% glutaraldehyde (Sigma-Aldrich Merck, St. Louis, MO, USA) for 18 h at 4 °C and dehydrated through a graded ethanol series (30–99.8%). Samples were then dried at the critical point of liquid CO_2_ using a Leica EM CPD300 automated critical point dryer (Leica Microsystems, Vienna, Austria) following the manufacturer’s “Bacteria” protocol. The dried samples were mounted on conductive carbon tape, sputter-coated with a 10 nm gold layer (CCU–010 HV, Safematic GmbH, Zizers, Switzerland), and examined at 5 kV using a secondary electron (SE) detector.

#### 2.4.5. Assessment of MONC Exposure on Bacterial Fatty Acid Composition (FAMEs)

The bacterial fatty acids were directly isolated from the cells treated with synthesized MONCs and the matrix itself after 24 h of exposure. For this purpose, the methodology described by Sasser [90] was applied to obtain fatty acid methyl esters (FAMEs). After treatment, 40 mg of bacterial biomass was taken from the culture, washed 3 times, and then saponified in NaCl:CH_3_OH:H_2_O_d_ (30 min, 100 °C) solution. Subsequently, the released fatty acids were methylated in HCl: CH_3_OH (20 min, 80 °C) and extracted into the organic phase of hexane: methyl tert-butyl ether solution (1:1). The washed FAME extract, prepared using saturated NaCl solution, was separated using an Agilent 7820A gas chromatograph (Agilent Technologies, Inc. Headquarters, Santa Clara, CA, USA) equipped with an FID detector and a capillary column (Ultra 2-HP, cross-linked 5% phenyl methyl silicone, 25 m, 0.2 mm and 0.33 film thickness), and analyzed using Sherlock Microbial Identification System software and the TSBA library (ver. 6.2B) (MIDI Inc., Newark, DE, USA).

### 2.5. Statistical Data Analysis

Raw data were statistically analyzed and are presented as the mean ± standard deviation (SD) based on at least three independent replicates, depending on methodological requirements, for each nanocomposite treatment. Statistical differences between MONC-treated samples and controls were evaluated using one-way analysis of variance (ANOVA), followed by Tukey’s honestly significant difference (HSD) post hoc test, with significance set at *p* < 0.05, and are indicated in the figures by different letters denoting statistically significant differences. Principal component analysis (PCA) and a loading plot approach were performed on spectroscopic infrared data transformed into second derivatives to identify similarities and differences in chemical composition and to qualitatively assess molecular interactions between MONCs and bacterial cells (ATR-FTIR). Integrated intensity analysis of specific infrared bands was used to construct box-and-whisker plots, allowing evaluation of the impact of nanocomposites on bacterial structure. PCA was also applied to FAME profiles, considering fatty acids with a relative contribution > 1.0% (*p* < 0.05), to detect shifts in fatty acid composition. Multivariate clustering of the MONC/NP dataset was performed to explore relationships between the toxicological index (MIC) and physicochemical properties. This comprehensive cluster analysis across all variables revealed statistical associations and inherent correlation patterns within the data. All statistical and graphical analyses were conducted using STATISTICA 13.3 (TIBCO Software Inc., Palo Alto, CA, USA), OriginPro 2023 (OriginLab Corporation, Northampton, MA, USA), and MS Office 2019 (Microsoft Inc., Redmond, WA, USA).

## 3. Results

### 3.1. Characterization of Newly Synthesized MONCs

**Figure 3:**
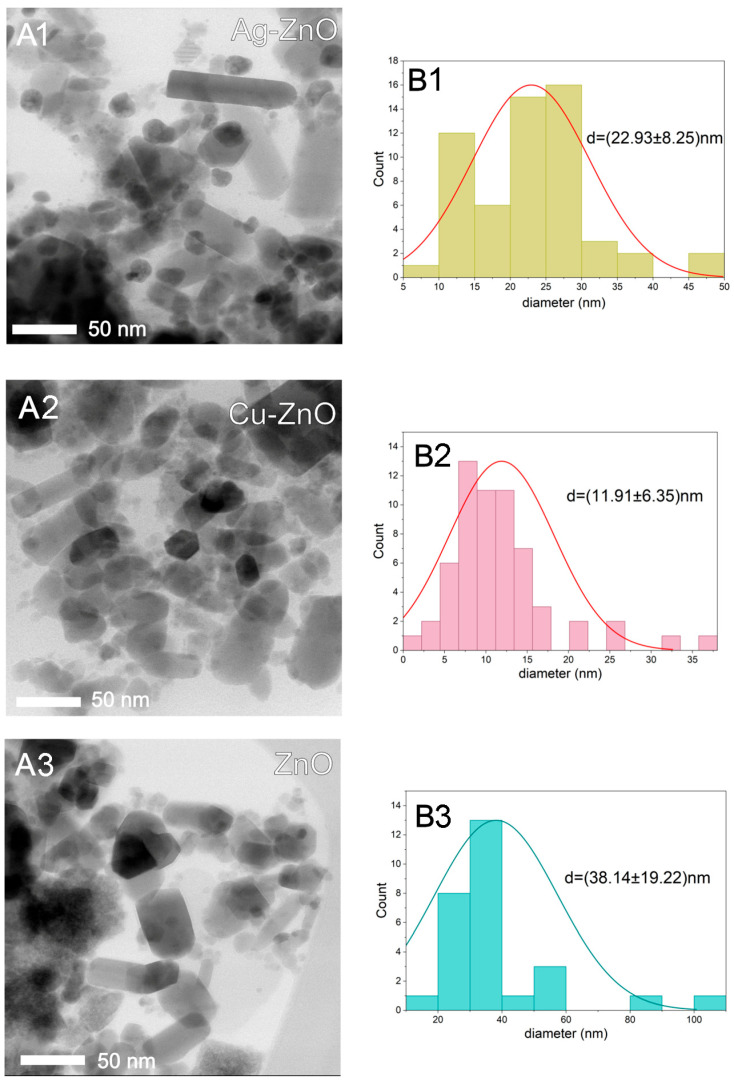
(**A1**–**A3**) Transmission electron microscopy (TEM) images of Ag– and Cu–doped ZnO nanocomposites and the ZnO nanoparticle matrix. (**B1**–**B3**) Particle size distribution of Ag and Cu nanoparticles within the ZnO matrix, and Feret’s diameter-based size distribution of the ZnO matrix particles.

Transmission electron microscopy (TEM) was used to assess the morphology and size distribution of the newly synthesized MONCs. TEM analysis revealed that both silver (Ag) and copper (Cu) nanoparticles, predominantly spherical in shape, were immobilized on the surface of the zinc oxide (ZnO) matrix (Figure 3). In contrast, ZnO nanoparticles exhibited an elongated or rod-like morphology, although some particles appeared irregular or barrel-shaped depending on their spatial orientation. Energy-dispersive X-ray spectroscopy (EDS) confirmed the elemental composition, indicating that silver accounted for 14.1 ± 2.7% of the Ag–ZnO nanocomposites, while copper contributed 9.5 ± 1.8% on the ZnO surface (Table 3). Using Feret’s diameter method, the average particle sizes were determined to be 22.93 ± 8.25 nm for Ag, 11.91 ± 6.35 nm for Cu, and 38.14 ± 19.22 nm for ZnO nanoparticles. These results show that Cu nanoparticles are approximately half the size of Ag nanoparticles. In contrast, the apparent size of ZnO particles, which form the primary matrix framework, is strongly influenced by their crystal orientation, making uniaxial size estimation challenging.

**Figure 4:**
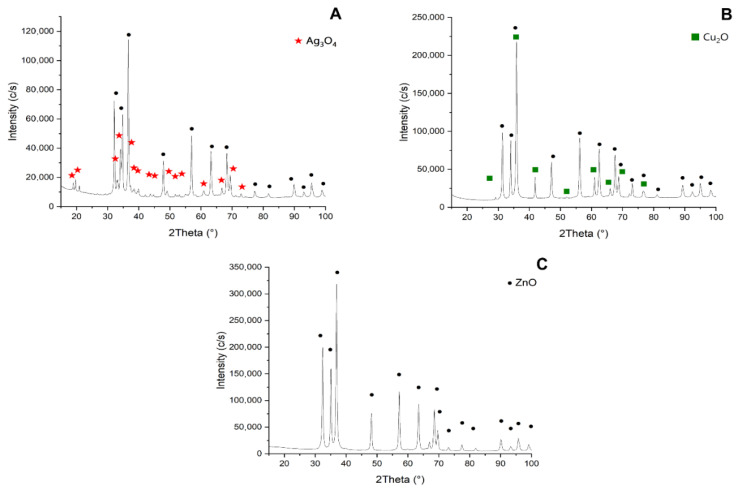
XRD analysis of MONCs doped with silver (Ag) and copper (Cu): structural properties and phase composition for ZnO-based MONCs (**A**,**B**) and ZnO NPs matrix (**C**). The dots on Figure 4A,B correspond to the matrix structure shown in Figure 4C.

X-ray diffraction (XRD) analysis was performed to determine the phase composition, crystallinity, and structural parameters of the MONCs and reference ZnO nanoparticles. The diffraction patterns confirmed high phase purity, with no detectable reflections corresponding to impurity phases (Figure 4).

The reference ZnO nanoparticles (Figure 4C) exhibited a single-phase hexagonal wurtzite structure (space group P6_3_mc, No. 186), with lattice parameters a = b = 0.32190 nm and c = 0.51490 nm, and an average crystallite size of 28.19 nm, serving as a structural baseline. Ag–ZnO nanocomposites (Figure 4A) also showed ZnO in the hexagonal wurtzite structure (P6_3_mc, No. 186), with expanded lattice parameters relative to the reference (Δa = 0.00343 nm, Δc = 0.00583 nm), indicating lattice distortion induced by the presence of silver species. Additional diffraction peaks were assigned to monoclinic Ag_3_O_4_ (space group P12_1_/c1, No. 14), with lattice parameters a = 0.35787 nm, b = 0.92079 nm, c = 0.56771 nm, and β = 106.14°. The corresponding crystallite sizes were 35.68 nm for ZnO and 16.19 nm for Ag_3_O_4_. Similarly, Cu–ZnO nanocomposites (Figure 4B) retained the hexagonal wurtzite structure of ZnO (P6_3_mc, No. 186), with lattice expansion relative to the reference (Δa = 0.00241 nm, Δc = 0.00527 nm), suggesting lattice strain associated with copper incorporation or interaction. Additional reflections were attributed to cubic Cu_2_O (a = 0.42700 nm), with crystallite sizes of 29.98 nm for ZnO and 35.12 nm for Cu_2_O.

The colloidal stability and surface properties of MONCs were evaluated via ζ-potential and DLS hydrodynamic diameter measurements at pH 6.5, using concentrations corresponding to optimal dispersion (Table 4). Pristine ZnO NPs had a positive ζ-potential of +12 mV. Cu–ZnO NCs were near neutral (−2.6 mV), while Ag–ZnO NCs showed a moderately negative ζ-potential of −22 mV. Hydrodynamic diameters reflected these trends: ZnO NPs had the largest aggregates (d = 2059.56 nm), Cu–ZnO NCs were slightly smaller (d = 1769 nm), and Ag–ZnO NCs had the smallest size (d = 1296 nm), consistent with enhanced electrostatic stabilization.

### 3.2. Antimicrobial Potential of Newly Synthesized MONCs

**Figure 5:**
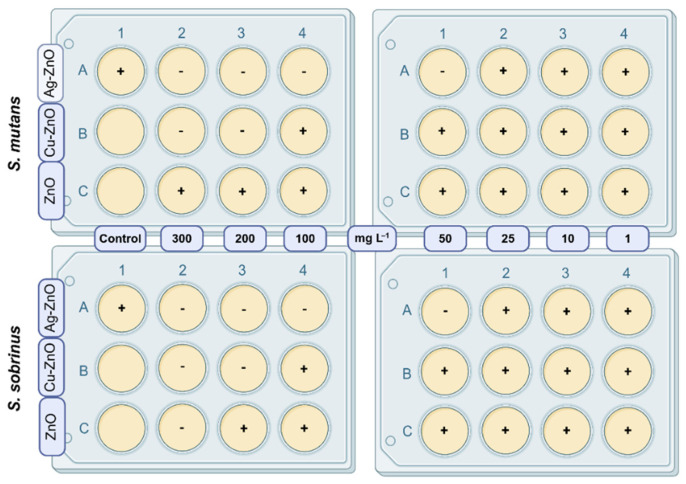
MIC values (mg L^−1^) for *Streptococcus* spp. strains treated with MONCs/NPs and untreated controls after 24 h. “+” indicates bacterial growth, “-” indicates the lack of growth, “no sign” refers to an empty well (no sample added). (Created in BioRender. Wasilkowski, D. (2026) https://BioRender.com/0zmdp5y, accessed on 14 April 2026).

The antibacterial activities of the newly synthesized MONCs and nanoparticles of the matrix itself (ZnO NPs) were assessed by determining the MIC. In this study, the model microorganisms—*Streptococcus* spp. strains, from the American Type Culture Collection (ATCC)*,* were chosen due to their important role in oral inflammation. The results revealed that both MONCs based on the ZnO matrix demonstrated antimicrobial properties and were dependent on the type of metal used (Figure 5). Among these materials, Ag–ZnO NCs exhibited the highest activity against *S. mutans* and *S. sobrinus*, with an MIC of 50 mg L^−1^. In contrast, Cu–ZnO NCs showed considerably weaker antimicrobial activity, with an MIC of 200 mg L^−1^. In turn, ZnO NPs used alone as the matrix did not exhibit any toxic effect against *S. mutans* at the highest tested concentration of 300 mg L^−1^; however, at 200 mg L^−1^, a growth-inhibitory effect was observed for *S. sobrinus*.

### 3.3. Ag^+^ and Cu^2+^ Ions Kinetics Release

**Figure 6:**
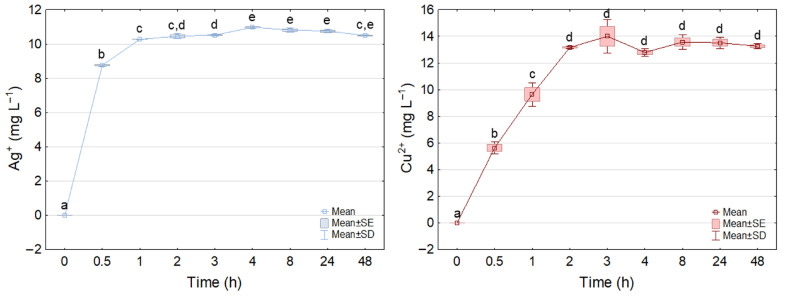
Release profiles of Ag^+^ and Cu^2+^ ions over 48 h from MONCs in BD 237500 medium for *Streptococcus* spp. Means sharing the same letter(s) are not significantly different (*p* < 0.05).

The release kinetics of Ag^+^ and Cu^2+^ ions from MONCs were investigated under conditions identical to microbiological assays, using BD 237500 medium for *Streptococcus* spp. and MONC concentrations corresponding to MIC values. As shown in Figure 6, Ag^+^ concentration reached 10.71 ± 0.08 mg L^−1^ after 4 h and remained stable up to 48 h, indicating attainment of equilibrium. In contrast, Cu^2+^ release reached a higher absolute concentration (14.00 ± 1.26 mg L^−1^) after approximately 3 h; however, due to the fourfold higher initial MONC concentration (200 mg L^−1^ vs. 50 mg L^−1^ for Ag–ZnO), the relative release was significantly lower. Specifically, Ag^+^ release corresponded to 21.42% of the initial dose, whereas Cu^2+^ accounted for only ~7%, indicating markedly different desorption efficiencies. Although Cu_2_O was identified by XRD, copper is expected to be released predominantly as Cu^2+^ under aqueous experimental conditions due to oxidation processes.

**Figure 7:**
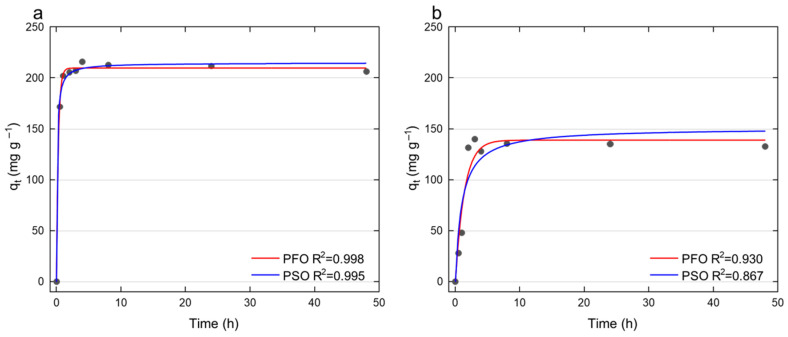
Desorption profiles of Ag^+^ (**a**) and Cu^2+^ (**b**) ions over 48 h from MONCs in BD 237500 medium for *Streptococcus* spp. fitted using PFO and PSO models.

Metal ion release kinetics were quantitatively evaluated using pseudo-first-order (PFO) and pseudo-second-order (PSO) models to elucidate the underlying mechanisms. For Ag^+^ ions, both models showed good agreement with experimental data (R^2^ > 0.90), with the PFO model providing the best fit (R^2^ = 0.998), indicating that the release process is predominantly governed by diffusion-controlled surface desorption. The high correlation and apparent maximum desorption capacity (q_max_ = 209.97 mg g^−1^) suggest that Ag^+^ ions are weakly bound to the ZnO surface and are readily released into the surrounding medium. In contrast, Cu^2+^ ion release showed lower conformity with the PFO model (R^2^ = 0.93) and a greater deviation from ideal kinetic behavior, indicating a more complex release mechanism. The lower apparent desorption capacity (q_max_ = 139.2 mg g^−1^) suggests stronger retention of Cu^2+^ within the ZnO matrix, likely due to partial lattice incorporation or stronger surface interactions. This behavior is consistent with a mixed mechanism involving both diffusion limitations and chemical interactions, as described by the PSO model (Figure 7).

**Figure 8:**
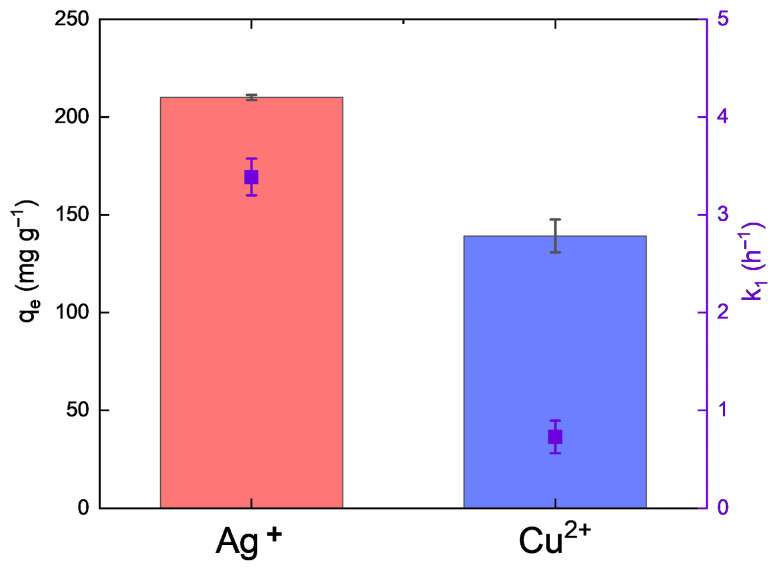
Summary of equilibrium release (q_e_) values (bars, left axis) and PFO model rate constants (k_1_) (markers, right axis) for the desorption of Ag^+^ and Cu^2+^ ions from MONCs. Error bars represent standard deviations.

Direct comparison of Ag^+^ and Cu^2+^ release under identical experimental conditions (Figure 8) further confirms that silver ions are released more rapidly and with higher efficiency than copper ions. Based on the pseudo-first-order kinetic constant (k_1_), corresponding to the time required to reach 63% of equilibrium concentration, Ag^+^ release reached equilibrium after approximately 28 min (k_1_ = 3.39 h^−1^). In contrast, Cu^2+^ ions required approximately 2 h 11 min (k_1_ = 0.727 h^−1^), indicating slower kinetics and stronger retention within the nanocomposite matrix. Although Cu^2+^ reaches its maximum concentration earlier due to higher initial loading, its release kinetics are slower, as indicated by the lower k_1_ value and reduced release efficiency.

### 3.4. Microbial-MONC Surface Chemical Interactions

**Figure 9:**
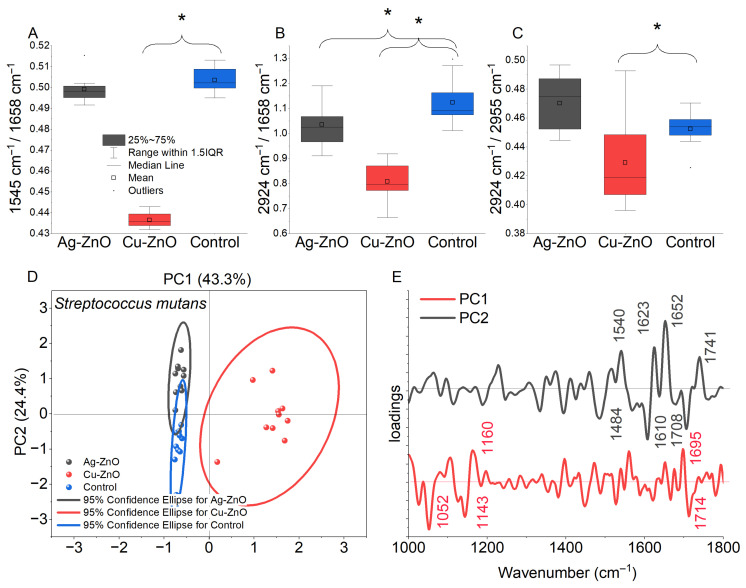
Box-and-whisker plots of protein and lipid-related biochemical parameters in *S. mutans* exposed to Ag–ZnO and Cu–ZnO NCs at the MIC compared with untreated control (**A**–**C**). Data are presented as medians with interquartile ranges and minimum and maximum values from vector-normalized infrared spectra. Statistical significance was assessed using the Mann–Whitney test, with * indicating *p* < 0.05. (**D**) PCA score plot based on vector-normalized spectra in the 950–1800 cm^−1^ range (95% confidence level). (**E**) PC1 and PC2 loading plots showing the spectral features responsible for group separation and associated biochemical variations.

FTIR analysis of *S. mutans* revealed composition-dependent biochemical responses to metal-loaded ZnO NCs. Cu–ZnO NCs induced pronounced changes (*p* < 0.05), notably in the integrated intensity I(1545 cm^−1^)/I(1658 cm^−1^) (amide II/I) ratio. Changes in I(2924 cm^−1^)/I(1658 cm^−1^) and I(2924 cm^−1^)/I(2955 cm^−1^) ratios indicated altered contributions of fatty acids/proteins and lipid chain composition (Figure 9A–C). Ag–ZnO NCs caused less pronounced but significant alterations (*p* < 0.05), primarily decreasing the I(2924 cm^−1^)/I(1658 cm^−1^) ratio. Both treatments affected carboxyl and phosphoryl groups, including nucleic acid phosphodiester bonds (1230 cm^−1^), as reflected in the I(1230 cm^−1^)/I(1540 cm^−1^) ratio. Opposite trends were observed in the I(1230 cm^−1^)/I(2924 cm^−1^) ratio: increased for Cu–ZnO and decreased for Ag–ZnO relative to the control (Figure S1A–C). PCA supported these observations: PC1 (43.3% variance) separated control from Cu–ZnO-treated samples, indicating extensive molecular alterations, while PC2 (24.4% variance) differentiated control from Ag–ZnO-treated samples and captured secondary Cu–ZnO effects. Together, PC1 and PC2 account for ~68% of spectral variability, highlighting the biochemical impact of the nanocomposites (Figure 9D). PCA loading plots identified the spectral features responsible for differentiating samples (Figure 9E). PC1 was dominated by negative contributions at 1708 cm^−1^ (C=O stretching in membrane lipids/fatty acids), 1610 cm^−1^ (β-sheet structures), and 1484 cm^−1^ (CH_2_ bending in lipids/amide II bands), explaining the main separation. PC2 showed negative loadings at 1714 cm^−1^ (C=O stretching in fatty acids, carboxylic acids, and ester groups), 1143 cm^−1^ (O–C–O asymmetric stretching of polysaccharides/nucleic acids), and 1052 cm^−1^ (C–O vibrations of carbohydrates), reflecting biochemical changes upon Ag–ZnO NC exposure.

**Figure 10:**
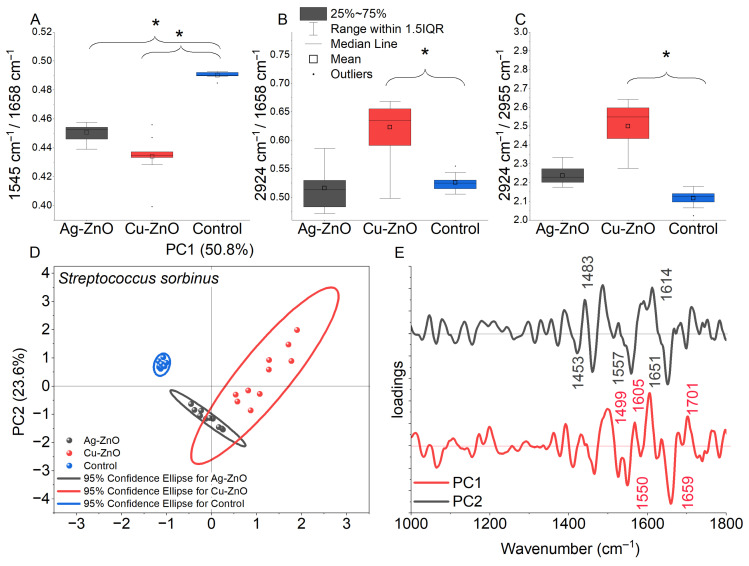
Box-and-whisker plots of protein and lipid-related biochemical parameters in *S. sobrinus* exposed to Ag–ZnO and Cu–ZnO NCs at the minimum inhibitory concentration (MIC) compared with untreated control (**A**–**C**). Data are presented as medians with interquartile ranges and minimum and maximum values from vector-normalized infrared spectra. Statistical significance was assessed using the Mann–Whitney test, with * indicating *p* < 0.05. (**D**) PCA score plot based on vector-normalized spectra in the 950–1800 cm^−1^ range (95% confidence level). (**E**) PC1 and PC2 loading plots showing the spectral features responsible for group separation and associated biochemical variations.

FTIR analysis of *S. sobrinus* revealed composition-dependent biochemical responses to metal-modified ZnO NCs. Cu–ZnO NCs induced pronounced changes (*p* < 0.05), notably in the integral intensity I(1545 cm^−1^)/I(1658 cm^−1^), I(2924 cm^−1^)/I(1658 cm^−1^), and I(2924 cm^−1^)/I(2955 cm^−1^) peak area ratios (Figure 10A–C), while Ag–ZnO NCs affected only the I(1545 cm^−1^)/I(1658 cm^−1^) ratio. In the phosphate region (1230 cm^−1^), Cu–ZnO significantly increased contributions of phosphate-containing compounds, whereas Ag–ZnO caused minor, insignificant deviations (Figure 10A). The I(1230 cm^−1^)/I(2924 cm^−1^) and I(1230 cm^−1^)/I(1540 cm^−1^) ratios indicated reduced DNA relative content, particularly for Cu–ZnO-treated cells (Figure S2A–C). PCA supported these findings: PC1 (50.8% variance) separated control from Cu–ZnO samples, showing extensive molecular alterations, while PC2 (23.6% variance) differentiated control from Ag–ZnO-treated cells and captured secondary Cu–ZnO effects. Together, PC1 and PC2 accounted for >74% of spectral variability (Figure 10D). PCA loading plots showed that PC1 was dominated by negative contributions at 1659 cm^−1^ (C=O stretching of α-helical amide I) and 1550 cm^−1^ (N–H bending/C–N stretching of amide II), reflecting protein backbone remodeling. PC2 featured negative loadings at 1614 cm^−1^ (β-sheet amide I) and 1481 cm^−1^ (β-structured peptide bonds, COO^−^ stretching, NH_3_^+^ deformation), highlighting nanocomposite-induced changes in protein structure and charge (Figure 10E).

### 3.5. Fatty Acid Profiling of Microbial Responses to Engineered MONCs

**Figure 11:**
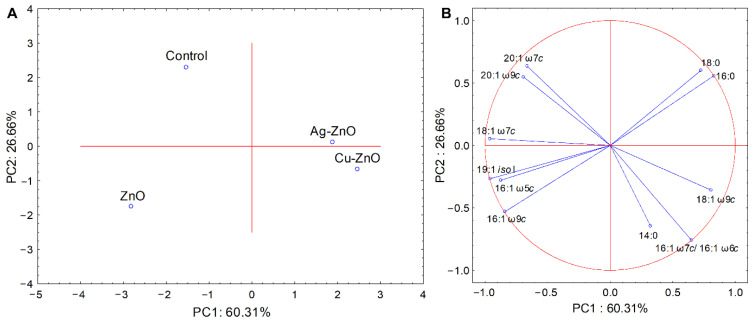
Projection of PCA analysis of FAME profiles profiles of treatment and control samples (**A**) and individual FAMEs (**B**) for *S. mutans* after 24 h of exposure to MONCs/NPs at MIC concentrations and in control.

**Figure 12:**
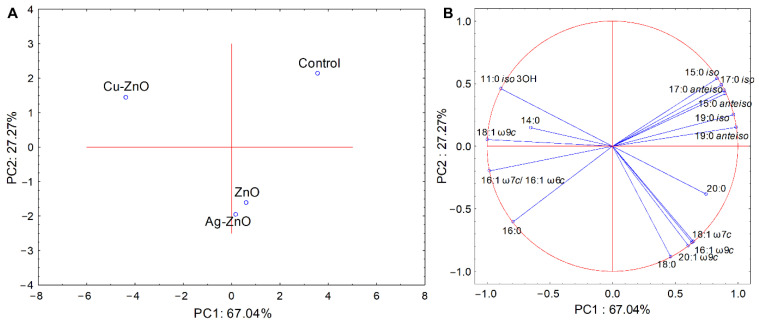
Projection of PCA analysis of FAME profiles of treatment and control samples (**A**) and individual FAMEs (**B**) for *S. sobrinus* after 24 h of exposure to MONCs/NPs at MIC concentrations and in control.

In this study, FAME analysis assessed the impact of MONCs on bacterial membrane composition and the effect of ZnO NPs. *Streptococcus* spp. treated with nanoproducts showed shifts in major saturated and unsaturated fatty acids compared to controls (Figure 11 and Figure 12). For *S. mutans*, PCA of FAME profiles revealed 60.31% and 26.66% variance, separating Ag–ZnO and Cu–ZnO NC-treated samples into a distinct cluster (PC1), while ZnO and control formed another group (Figure 11A). MONC treatment primarily affected unsaturated fatty acids 18:1 ω7c, 18:1 ω9c, and 20:1 ω9c, whereas ZnO NPs also influenced 16:0, 18:0, 18:1 ω9c, and 20:1 ω9c (Figure 11B, Table S1).

The analysis of fatty acid composition in *S. sobrinus* reveals that MONCs and released ions altered membrane composition (Figure 12A). PCA (PC1 67.04%, PC2 27.27%) identified three groups: Cu–ZnO, Ag–ZnO NCs with ZnO NPs, and control. MONC treatment reduced branched-chain fatty acids (15:0, 17:0, 19:0 *iso*/*anteiso*) and affected 16:0. Metal-specific effects were observed: 19:0 *anteiso* doubled with Ag^+^, 18:1 ω9c doubled with Cu^2+^, and 11:0 iso 3OH appeared only in Cu–ZnO-treated cultures (2.66%) (Figure 12B, Table S2).

### 3.6. SEM Imaging of Cell-MONC Effect

**Figure 13:**
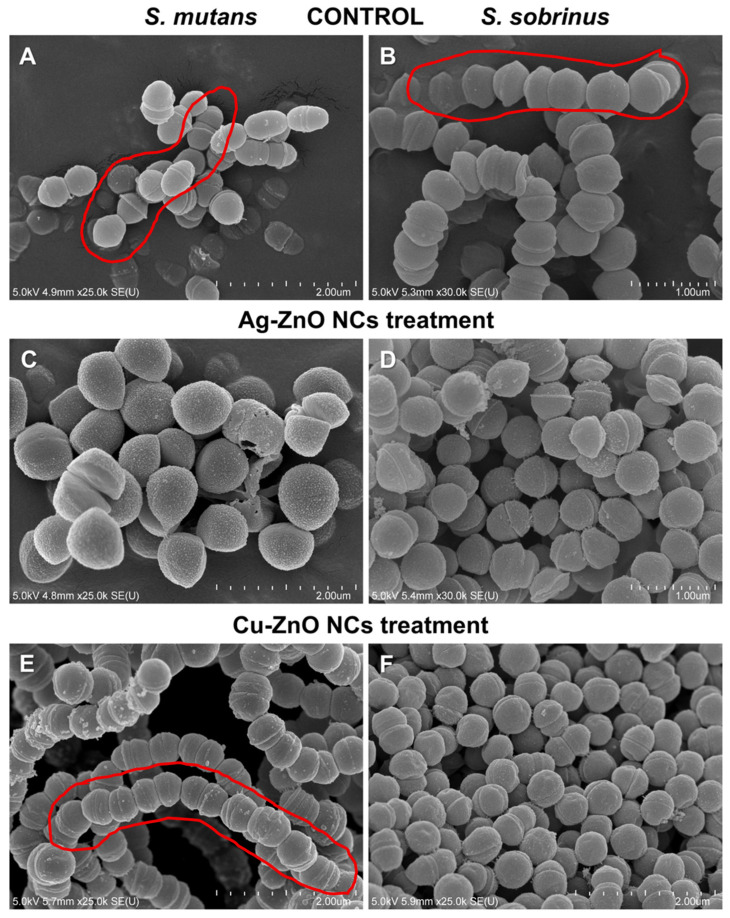
SEM micrographs of *S. mutans* and *S. sobrinus* in untreated control (**A**,**B**) and after treatment with Ag^+^ (**C**,**D**) and Cu^2+^ (**E**,**F**) MONCs. Red loops indicate chain-like structures formed by the cells.

Scanning electron microscopy was used to visualize the bacterial surface morphology after Ag–ZnO and Cu–ZnO NC treatment compared with non-treated control cells. The micrographs obtained reveal the destructive impact of the tested MONCs on bacterial cells after 4 h of exposure (Figure 13). A prominent cytotoxic effect observed was the leakage of cytoplasmic content through membrane disruptions at sites of direct contact with the Ag–ZnO NC (Figure 13C,D). The consequence of disrupting the continuity of the wall and cell membrane structure is, according to the control cells, also accompanied by cell maceration. Furthermore, a loss of the ability of *S. mutans* and *S. sobrinus* to form chain or cluster structures was observed in the culture with Ag–ZnO NCs (Figure 13C,D), whereas this phenomenon in Cu–ZnO NC treatment was observed only in *S. sobrinus* culture (Figure 13F). It is also worth noting a slight deformation of the cell surface only of *S. sobrinus* in the presence of Cu–ZnO NCs was noted (Figure 13E,F). Furthermore, treatment with Ag–ZnO NC resulted in an increased volume of *S. mutans* cells as compared to control (Figure 13C).

### 3.7. Multivariate Analysis of MIC in Relation to Physicochemical Properties of MONCs and NPs

**Figure 14:**
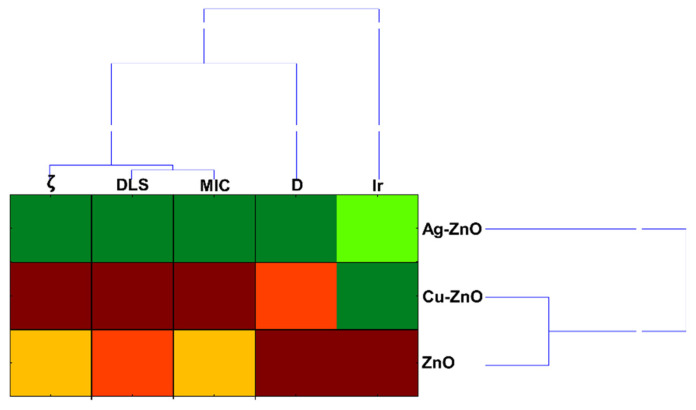
Visualization of ζ-potential, Dynamic Light Scattering (DLS), Minimum Inhibitory Concentration (MIC), crystallite size (D), and ion release (Ir) via cluster analysis, revealing prominent agglomeration trends in the heatmap under Ag–ZnO NCs, Cu–ZnO NCs, and ZnO NPs matrix stress. Each color represents a different cluster, grouping together objects that are more similar to each other than to those in other clusters.

A multivariate analysis was performed to assess the effects of Ag–ZnO, Cu–ZnO, and the ZnO matrix on the variability of both *Streptococcus* strains, as well as on the relationship between the toxicological index (MIC) and the physicochemical properties of the MONC/NP dataset. Furthermore, a cluster analysis encompassing all variables was conducted to verify their statistical associations and identify any correlation patterns present in the dataset. The performed cluster analysis for *S. mutans* and *S. sobrinus* exposed to MONCs and NPs revealed that the most differentiating were Ag–ZnO NCs, while Cu–ZnO NCs and matrix ZnO NPs had a comparable impact on both bacteria (Figure 14). Moreover, it was found that iron release and crystallite size had a major discriminating influence on the obtained dendrogram projection. It is also worth noting that MIC, representing the biological parameter, was characterized by a strong positive correlation with ζ-potential and DLS (r = 0.999, *p* < 0.05). In contrast, it was negatively correlated with Ir (r = −0.999, *p* < 0.01).

## 4. Discussion

### 4.1. Evaluation of Novel MONCs Obtained by Chemical Synthesis

Implants are commonly used in modern medicine. However, their long-term functionality is often affected by microbial colonization and the formation of biofilms. It can lead to serious infections and impact failure. Preventing bacterial adhesion and growth on implant surfaces, therefore, is a critical challenge in clinical practice. To respond to this problem, researchers are looking into materials with vast antimicrobial potential. Among many engineering nanomaterials, particularly silver (Ag) and copper (Cu), have received significant attention due to their well-documented ability to inhibit and eliminate pathogenic microorganisms [30]. In this study, soft chemical hydrolysis with polycondensation was used for synthesis. Metal nitrate (AgNO_3_)and acetate (Cu(CH_3_COO)_2_·H_2_O) salts were used as the precursors of metal NPs and a ZnO NPs matrix. This process produced two types of metal oxide nanocomposites (MONCs): Ag_3_O_4_-ZnO (Ag–ZnO) and Cu_2_O-ZnO (Cu–ZnO). Moreover, integrating metallic nanoparticles with ZnO makes it a suitable matrix for systems used in biological applications due to their high biocompatibility, low cytotoxicity, and affordability [91,92].

The fundamental step in nanotoxicological and biomedical research is the detailed characterization of nanoproducts, including physicochemical and morphological properties such as size, structure, chemical composition, shape, and surface charge. A crucial factor influencing the functionality of NPs is their tendency to form aggregates [30,31]. The in vivo aggregation behavior of nanoparticles remains uncertain and may evolve. Moreover, particles near implants are unlikely to remain uniformly monodisperse and stable [93]. Therefore, for each experiment, the aqueous working solution of MONCs was sonicated before usage to minimize this phenomenon, in accordance with the approach used in the studies by Long et al. [94] and McQuillan et al. [95].

In this study, TEM was used to analyze the morphology and size of the newly synthesized MONCs as well as their composition. The obtained nanoproducts meet the criteria for nanoparticles as defined by the European Commission Recommendation 2022/C229/01 [96], where 50% or more of their particles have an external dimension of 1–100 nm. According to the TEM micrographs, the ZnO NCs were rod-liked shape, exhibiting a higher efficiency of Ag^+^ ion immobilization than Cu^2+^ on the matrix surface. XRD analysis provides more detailed information about the structure and size of MONCs. This analysis confirmed that the final products are stable and of high purity. After Ag–ZnO NC synthesis, silver oxide Ag_3_O_4_ (21.3%) was formed, which is in accordance with Al-Gaashani et al. [97], with a crystallite size of 16.19 nm. In turn, Cu ions phase into ZnO NCs, forming Cu_2_O, which is measured to be 35.12 nm crystallite size. Overall, the XRD results demonstrate that Ag– and Cu–modified ZnO systems preserve the wurtzite ZnO framework while introducing secondary metal oxide phases and measurable lattice distortions, reflecting strong interfacial interactions that are expected to influence the functional properties of the nanocomposites. It is also worth noting that the crystallite size of the ZnO NP matrix was consistent with the SDS card data, while XRD analysis confirmed their hexagonal structure. For example, Ag–ZnO NMs synthesized by Abdel Messih et al. [98] through a combined chemical reduction method yielded larger products than in this study, crystalline particles measuring 47.5–61 nm and with a hexagonal ZnO shape, while Al-Gaashani et al. [97] reported particle sizes in the range of 60.20–74.09 nm using Scherrer modeling. The hexagonal Ag–ZnO nanoproducts obtained using the starch-mediated method, as applied by da Silva et al. [99], were characterized by a crystallite size of 13.9–16.5 nm, which is in accordance with the product in this study. Similarly, Khatir and Zak [100] used Cu NMs with a crystallite size of 18 nm, doped in a spherical ZnO matrix (approximately 40 nm). The ultrasonic-assisted method was employed to fabricate Cu–ZnO NMs with an average size of 34 nm and unspecified shape [101].

Surface charge is one of the crucial physicochemical parameters strongly influencing biocompatibility and cellular interactions of nanoscale materials. The ζ-potential, as a critical factor in nanomedicine design, indicates how nanoparticles are internalized and processed in biological systems. The surface potential of nanomaterials affects their interactions with cells, proteins, and biological environments, thereby influencing their biocompatibility and uptake in medical applications [102,103]. Moreover, negatively charged nanomaterials can improve stability and reduce non-specific interactions, which often leads to improved biocompatibility and, consequently, better suitability for medical applications. This phenomenon is associated with the higher affinity of positively charged particles for negatively charged cell membranes, causing them to interact more strongly with human cells and potentially leading to cytotoxic effects [102,104]. In this study, a ZnO NP matrix was defined by imparting a positive ζ-potential, which can be attributed to protonated surface hydroxyl groups (Zn–OH_2_^+^/Zn–OH) under slightly acidic conditions. The largest hydrodynamic diameter, indicating a tendency towards extensive agglomeration in the aqueous suspension, is consistent with its relatively low surface charge. Among both MONCs, the highest ζ-potential was observed for Ag–ZnO NCs (−22 mV), indicating high stability and a substantial change in surface chemistry, most likely arising from the presence of silver-containing species, altered surface hydroxylation, and/or adsorption of anionic groups. A similar trend was observed by Dutta et al. [105], Ramakrishnegowda et al. [106], and Efthimiou et al. [107]. In contrast, Wang et al. [108] presented Ag/ZnO characterized by a lower ζ-potential compared to our results. In turn, the Cu–ZnO system exhibited a ζ-potential close to neutral, suggesting partial charge compensation at the ZnO surface and significantly weaker electrostatic stabilization. Moreover, such near-neutral ζ-potential values typically favor particle–particle interactions. In turn, this negative surface charge enhances electrostatic repulsion between particles and is generally associated with improved colloidal stability. These trends clearly indicate distinct interaction mechanisms of Ag^+^ and Cu^2+^ with the ZnO NPs carrier. A comparable potential as an ion carrier has been reported for a widely used material, TiO_2_ nanoparticles, which serve, among others, as a matrix for Ag^+^ and Cu^2+^ ions. These systems produce nanomaterials with a negative surface potential, as reported, for example, by Baydar et al. [109] and Jariyaboon et al. [110] for Ag–TiO_2_ NMs, as well as by Gan et al. [111] and Ning et al. [112] for Cu–TiO_2_ NMs, who observed negative ζ-potential for metal oxide nanomaterials with TiO_2_, making our MONCs suitable for applications, including those in medicine. This result indicates that Ag incorporation most effectively suppresses particle agglomeration among the investigated systems. Notably, all measured hydrodynamic diameters are substantially larger than the crystallite sizes derived from XRD, confirming that the nanoparticles exist as secondary agglomerates in aqueous media rather than as isolated primary nanocrystals. All dispersions maintained a slightly acidic pH, demonstrating that neither the ZnO matrix nor the Ag– or Cu–modified nanocomposites significantly altered the acidity of the medium. This stable pH environment likely governs the surface protonation state and, consequently, the observed ζ-potential values and aggregation behavior. Overall, the combined ζ-potential and hydrodynamic diameter analyses demonstrate that Ag^+^ incorporation significantly enhances the colloidal stability of ZnO-based nanocomposites. In contrast, Cu modification leads to near-neutral surface charge and a higher propensity for aggregation, highlighting the critical role of metal identity in tuning the interfacial and colloidal properties of ZnO nanomaterials.

The inhibitory mechanism of nanoparticles against bacteria depends on the release of active metal ions that attack the biological molecules (membrane proteins, lipids, polysaccharides) and cover the cell surface, forming a molecular layer due to the high surface reactivity [18]. This interaction initiates pore formation in cell membranes and cell wall damage, causing translocation and internalization of NP ions inside the cell, starting the interruption and damage of DNA and ATP or generating reactive oxygen species (ROS), disrupting cellular structure and cell cycle, interfering with physiological processes, and leading to inhibition of cell growth [20,21,22]. In this study, the release of Ag^+^ and Cu^2+^ ions was estimated using adsorption kinetic models, specifically the PFO and PSO expressions, for *Streptococcus* spp. medium. Culture media were selected for this study, as the composition of the medium strongly influences ion release kinetics and may alter the behavior of NPs in dispersion, and is therefore critical for accurately reflecting experimental conditions [113]. The obtained data indicate that Ag^+^ ions in the BD medium were released more rapidly, reaching near-equilibrium within approximately 30 min, and attaining a maximum concentration after 4 h of incubation. In contrast, Cu^2+^ ions reached their maximum concentration earlier (after approximately 3 h); however, their overall release was less efficient. These results suggest that, although surface diffusion contributes to ion release, Cu^2+^ ions experience stronger interactions with the composite matrix, which limit their desorption. The apparent desorption capacity, based on the PFO model, was 209.97 mg g^−1^ for Ag^+^ and 139.2 mg g^−1^ for Cu^2+^ ions. The maximum Ag^+^ concentration corresponded to 21.42% of the initial MONC dose, whereas the released Cu^2+^ ions represented only ~7% of the initial Cu–ZnO nanocomposite concentration. Only a limited number of studies addressing nanoparticle toxicity have quantitatively evaluated the release of active ions in biologically relevant media, despite their key role in determining the functional and antimicrobial properties of such systems. In the study by McQuillan et al. [95], the rate of dissolution for the Ag NPs in the modified Luria–Bertani (LB) medium (100 µg mL^−1^) was determined. The sample was next sonicated, giving an initial Ag^+^ dose of 134.49 ng g^−1^, which reached a maximum of 440 ng g^−1^ after 24 h at 37 °C and with gentle shaking (100 rpm). These results suggest a more favorable approach for achieving efficient Ag^+^ ion release, comparable to the levels observed in the present study. In another study, Long et al. [94] investigated the release of Ag ions in NaHCO_3_ medium (Sinopharm) for *E. coli* in toxicological experiments, observing the highest ion release from an initial concentration of Ag NPs coated with sodium citrate (15 mg L^−1^), reaching 68.9 ng mL^−1^ after 24 h. The release of copper ions from nanoparticles in different media has been considerably less explored than that of silver. Nonetheless, a few experimental studies have addressed this issue. Among them, Reyes et al. [114] investigated algal growth in tris–acetate–phosphate medium using *Chlamydomonas reinhardtii* exposed to Cu NPs at an initial concentration of 20 mg L^−1^ and 37 °C. After 96 h of incubation, the concentration of released Cu ions ranged from 0.4 to 13.15 mg L^−1^. This substantial difference confirms that Ag^+^ desorption is more dynamic and kinetically favorable than Cu^2+^ release. The observed disparities in ion release behavior can be rationalized by differences in metal–carrier interactions and colloidal properties of the MONCs. Ag ions are known to form weaker surface interactions with oxide matrices compared to Cu ions, facilitating their faster desorption. Additionally, the smaller hydrodynamic diameter and more negative ζ-potential of the Ag–ZnO NCs enhance electrostatic repulsion and ion mobility, promoting Ag^+^ release into the medium. In contrast, the larger hydrodynamic diameter and near-neutral ζ-potential of Cu–ZnO indicate higher colloidal stability and stronger copper binding, which collectively hinder Cu^2+^ desorption and prolong the time required to reach equilibrium.

### 4.2. Investigation of the Antimicrobial Properties of Synthesized MONCs

Engineered nanoparticles are a useful and promising tool against bacterial pathogens, particularly those resistant to antibiotics. Among them, Ag and Cu NPs are characterized by efficient antimicrobial activities. Similarly, ZnO NPs exhibit a wide spectrum of activity against microorganisms [29]. Therefore, in this study, metal ions doped into ZnO NPs were used as a matrix to synthesize nanoproducts as promising antibacterial tools.

Currently, studies are increasingly being conducted in the field of nanotoxicology to assess the potentially toxic effects of nanostructures on microorganisms. In such studies, reference bacterial strains are most commonly used. Accordingly, the effects of engineered nanocomposites, obtained through chemical synthesis, on two reference bacterial strains, *Streptococcus mutans* and *S. sobrinus*, were tested. These bacterial strains are among the primary microbial contributors to dental caries, exhibiting cariogenic potential through their ability to adhere to tooth surfaces, synthesize extracellular polymeric substances (EPS), and promote biofilm formation [3,4].

The results of this study highlight that metal NPs combined with a ZnO matrix exhibit the strongest antimicrobial activity. Among these, Ag–ZnO NCs demonstrated the highest effectiveness against the tested bacterial strains. This may appear counterintuitive because Gram-positive bacteria are able to oxidize Cu NPs more easily and faster than Ag NPs on the cell surface [115]. This can be explained by silver’s interaction with dissolved O_2_ in media, which oxidizes metallic silver and produces Ag^+^ ions that act as active antimicrobial agents [116]. Notably, Cu–ZnO NCs also exhibited high activity towards both *Streptococcus* spp. strains, but at a concentration four times higher than that of Ag–ZnO NCs. Unfortunately, the weak antibacterial effect of Cu–ZnO (−2.8 mV) NCs can be attributed to the formation of larger agglomerates and reduced surface and contact with the cells. Moreover, the low value of ζ-potential between −30 and 30 mV is considered to indicate lower reactivity and toxicity of metal NPs [117]. DLS of synthesized MONCs in the media solution distinguished Cu–ZnO NCs with the largest hydrodynamic size, which reduced their reactivity, confirming lower antimicrobial properties of Cu–ZnO than Ag–ZnO NCs. This situation can be attributed to the neutral pH of bacterial culture media, which reduces the dissolution of copper oxide and the release of Cu^2+^ or Cu^+^ ions [118]. The dependency between the culture media and the level of Cu ion release was studied by Käkinen et al. [119], who observed five times lower Cu^2+^ ion concentration in M9 mineral media than in those supplemented with amino acids and NaCl. Additionally, this phenomenon can be attributed to the reaction of Cu^2+^ ions with Cl^−^ ions in the medium [120]. The present research distinguished ion release and crystallite size as the primary factors driving the observed differences among the samples.

Although several studies have reported the synthesis and antimicrobial properties of Ag– and Cu–ZnO NCs, research on their effectiveness against oral pathogenic bacteria remains limited. Moreover, only a minority of studies to date have focused on using ZnO NPs, rather than the commonly employed TiO_2_ NPs, as a matrix for antimicrobial ions such as Ag and Cu in medical applications. A case in point is the study of Wang et al. [108], revealing the toxic effect of Ag/ZnO nanocomposite against *S. mutans* at the concentration of 256 µg mL^−1^, i.e., lower than in the present study. For comparison, a nanoproduct with greater efficacy than that reported in this study was achieved against *S. mutans* at a lower concentration, MIC = 25 µg mL^−1^ [121] and MIC = 31.25 µg mL^−1^ for NC modified with reduced graphene oxide [122]. In contrast, at half the MIC (128 µg mL^−1^), no difference in viability was observed compared with the control [123]. Also, similar effectiveness according to our observations in the case of *S. sobrinus* treated with Cu–ZnO NC was achieved by Sevinç and Hanley [124] with a MIC concentration of 50 µg mL^−1^. Interesting results obtained by Ramazanzadeh et al. [125], where NPs CuO–ZnO coated brackets caused no colony growth of *S. mutans* after two hours of exposure. The studies conducted in this area have primarily focused on the evaluation of ZnO nanomaterials with antibacterial potential against *E. coli* and *S. aureus*. For instance, Ag/ZnO and Cu/ZnO NCs, as 1% concentration treatment, showed a reduction in the growth of *S. aureus*, with inhibition rates of 92.2% and 62.2%, respectively [126]. Ramakrishnegowda et al. [106] reported promising results of the biocompatible nanocomposite AgO–Ag–ZnO with strong antimicrobial activity, inhibiting growth in the following order: *E. coli* (Gram-negative) > *S. aureus* (Gram-positive) > *B. cereus* (Gram-positive) (most resistant). Cu NP-doped ZnO nanocomposites have also been studied as antimicrobial agents. For instance, the Cu@ZnO nanocomposite reported by Medina-Ramírez et al. [127] exhibited antibacterial activities for the very high applied NP doses of 750 mg L^−1^ in the case of *E. coli* and 500 mg L^−1^ for *S. aureus*, which may pose a concern about safety for the environment. In this study, the tested Cu–ZnO NCs were characterized by better antimicrobial activities for the lower NC concentrations against *S. mutans* and *S. sobrinus* (MIC = 200 mg L^−1^). Also, Tsogoo et al. [128] applied 2 × 10^−7^ g mL^−1^ of Cu–doped ZnO NPs onto bacteria-seeded Petri dishes, observing after 24 h of incubation 91.3–98.9% inhibition of *E. coli* colonies and 67.2–97.4% inhibition of *S. aureus* colonies. The antimicrobial potential of Ag– and Cu–ZnO NCs has also been demonstrated against extensively drug-resistant bacterial strains, including *Mycobacterium tuberculosis*, *E. coli* (rifampicin-resistant), *S. aureus* (methicillin-resistant), *K. pneumoniae,* and *Salmonella enterica*, by Sukri et al. [129], Govindasamy et al. [130], and Heidary et al. [131]. The promising antipathogenic properties exhibited by the synthesized MONCs against strains responsible for oral infections suggest that the present results provide a promising basis for further research on the application of these materials as antimicrobial agents for preventing oral infections.

It is also worth noting that the metal oxide matrix ZnO NPs generally had no toxic effect on the tested microorganism. However, these findings are contrary to the prevailing literature trends, as ZnO NPs are widely recognized for their antibacterial properties while simultaneously being non-toxic to human cells and exhibiting good biocompatibility [92]. An example of the adverse effects of ZnO nanoparticles on bacterial cells was provided by Jowkar et al. [91] against *S. mutans*. In contrast, higher sensitivity of *B. cereus* to ZnO NPs was observed by Esmailzadeh et al. [132] and Krzepiłko and Matyszczuk [133], and can be attributed to the presence of phosphodiester bonds between teichoic acid in the thick peptidoglycan layer, which makes the cell surface more attractive for positively charged ions [132].

In toxicology, to understand the interactions of NPs with biological systems, it is necessary to know their physicochemical properties, including surface charge, which is particularly responsible for NPs adhering to bacterial cell surfaces [79]. The adhesion of NPs to the bacterial surface can then lead to the formation of cell cavities, destabilization of the outer layer, and changes in the composition of functional groups [134]. The combined effect of mechanical and microbial properties of NPs can result in the incorporation of NPs into the bacterial surface, which is regulated by the peptidoglycan thickness, turgor pressure maintenance, and mechanical properties [135]. Furthermore, copper nanoparticles exhibit enhanced activity against Gram-positive bacteria and can also induce biofilm dispersion or inhibit biofilm formation [115]. The SEM micrograph in our study provides evidence that the synthesized MONCs cause destruction of the Gram-positive bacterial surface integrity by binding to thiol, sulfur, and phosphorus groups, resulting in visible membrane disruptions that lead to membrane perforation and subsequent leakage of intracellular contents [116]. Moreover, treatment with Ag–ZnO NCs impaired the ability of both bacterial strains to form chain or cluster structures, whereas this phenomenon was observed only in the *S. sobrinus* culture with Cu–ZnO NC treatment. Consistent with our results, ZnO-based matrix combined with a zeolite nanocomposite (ZnONC–CS) induced a reduction in *S. mutans* biofilm formation [4]. In other studies, Ag–ZnO NC inhibited biofilm formation by both the Gram-negative *P. aeruginosa* and Gram-positive *S. aureus* [136] and by *S. mutans*, achieving 69% inhibition [123]. Also, Wang et al. [108] and Wu et al. [122] confirmed proliferation and maceration of the cell surface as well as lack of biofilm formation in *S. mutans* culture treated with Ag/ZnO NCs.

Despite extensive investigation, the influence of elemental composition and atomic chemical states of the prepared nanoparticles on their antimicrobial activity remains unexplored. For this reason, we performed a detailed analysis of NCs with bacterial cell interactions at the level of chemical functional groups using FTIR spectroscopy as well as bacterial FAME profiling. The analyses for *S. mutans* and *S. sobrinus*, representing a Gram-positive bacterial strain, demonstrated biochemical responses dependent on both composition and chemical functional groups in the presence of metal-loaded ZnO NCs. In the case of Cu–ZnO NC treatment, structural alterations in protein conformation likely caused by interactions between the bacterial cell wall and NCs were observed, especially a shift in protein secondary structure, particularly within the amide II and amide I regions. This tendency was confirmed by Thakur et al. [137], who observed similar absorption peaks for produced CuO and Cu–ZnO NCs. Furthermore, membrane lipid reorganization, reflected by alterations in fatty-acid chain length, saturation level, and branching, indicated a substantial disruption of lipid membrane integrity [86,138]. For comparison, the results obtained for Ag–ZnO NCs indicated a reduced relative DNA contribution, consistent with previous FTIR-based studies on nucleic acid–protein discrimination in microbial systems, along with divergent changes in phosphate-containing compounds and saturated fatty acids, suggesting fundamentally different biochemical interactions for copper- and silver-modified ZnO NCs [139,140]. In the present study, Ag–ZnO-induced perturbations in *S. mutans* were observed in relation to carboxyl and phosphoryl functional groups, including the phosphodiester bonds of nucleic acids. In contrast, in *S. sobrinus* cultures, the relatively weaker impact on lipid-associated structures, along with slightly elevated values, indicates an enhanced contribution of phosphate-related compounds associated with a stress-response mechanism. Overall, the FTIR band-ratio analysis confirms distinct molecular targets and mechanisms of interaction for Cu–ZnO and Ag–ZnO NCs in *Streptococcus* spp., which act through distinct molecular pathways and lead to different patterns of biochemical responses, primarily related to proteins and phosphate-containing compounds, likely reflecting stress-induced remodeling and/or changes in membrane fluidity. Both sets of experiments showed that MONCs altered the bacterial FAME composition compared to the control, with changes in the percentage of monounsaturated fatty acids, specifically C18 and C20 fatty acids, in the *S. mutans* membrane. This finding is in accordance with the FTIR results, which showed a prominent loading at 1328 cm^−1^ for CH_3_ groups. In turn, a quite different situation was observed for *S. sobrinus*, where the MONCs treatment resulted in a decrease in the percentage of branched-chain fatty acids (C15, C17, and C19) for both *iso* and *anteiso* conformations, accompanied by an increase in 18:1 ω9c. This effect was more pronounced under Cu–ZnO NCs treatment. The antibacterial activity of NPs is largely dependent on the release of active metal ions from the NCs. The first line of defense against microbial stressors, which are present in the cellular environment, including NPs and ions, constitutes the surface layer of cells. Its components, such as membrane proteins, lipids, and polysaccharides, due to their high reactivity, can form a molecular surface layer with NPs [18]. This effect is characteristic, especially for a positive microbial surface charge and a metal ion core, because of electrostatic interactions [57]. Among the synthesized MONCs, the Ag–ZnO NCs variant showed the highest efficiency against bacterial cells. This trend suggests that increased NPs attachment efficiency is associated with bacteria possessing more negatively charged surfaces, likely because the stronger electrostatic attraction enhances binding between Ag ions and electron-donating chemical groups, such as microbial membranes and proteins (sulfhydryl, amino, phosphate, or carbonyl) [56]. Moreover, in the presence of Ag NPs, stressors can increase resistance mechanisms through significant structural changes in the peptide and amino acid regions or the amid composition in bacterial cells [141]. Unfortunately, chemical-level investigations of NCs–cell interactions are rarely used to assess changes in the composition of specific chemical groups associated with these interactions. Such an approach seems particularly justified and necessary in the context of the deliberate use of nanomaterials, especially with respect to their targeted applications, for instance in nanomedicine. For example, the treatment of *S. aureus* with Ag and Cu NPs, as revealed by FTIR spectroscopy, showed a decrease in the amid (I and II) bands and CH_2_ groups, resulting in an increase in membrane fluidity [142]. In turn, Metryka et al. [79] reported that treatment of *B. cereus* and *S. epidermidis* with Ag^+^ NPs induced shifts in protein structure (1545/1658 cm^−1^) and phosphate-containing compounds (1240 cm^−1^). These observations are consistent with our results for *S. sobrinus* in the presence of Ag MONCs. In accordance with our study, Metryka et al. [72] reported that the treatment of *S. epidermidis* with Cu^2+^ nanomaterials resulted in a decrease in the content of unsaturated fatty acids. Rogowska et al. [143] observed an increase in the levels of free fatty acids in *E. coli*, *K. pneumoniae*, and *S. epidermidis* due to ZnO NPs stress, which may also indicate cell membrane degradation resulting from the breakdown of long-chain phospholipids.

## 5. Conclusions

Antimicrobial resistance (AMR) represents a critical global health challenge due to the increasing prevalence of multidrug-resistant bacterial strains. Engineered nanoparticles have emerged as a promising strategy to combat antibiotic-resistant pathogens and pathogenic biofilms, including those implicated in oral diseases, which remain a significant public health concern. In this work, nano-scale metal oxide materials incorporating Ag^+^ and Cu^2+^ ions on a ZnO matrix were successfully synthesized using the chemical reduction method. The synthesized nanocomposites were characterized using TEM, XRD, ζ-potential, DLS, and pH measurements, confirming that the obtained nanoproducts fulfill the criteria for nanomaterials. Moreover, these analyses provided evidence of successful coating of Ag and Cu NPs on the ZnO NPs matrix and demonstrated that the final products Ag_3_O_4_-ZnO (Ag–ZnO) and Cu_2_O-ZnO (Cu–ZnO) are stable and pure.

The obtained nanocomposites exhibited significant antimicrobial activity. Among them, Ag–ZnO NCs showed the highest efficacy against the tested bacterial strains (MIC 50 mg L^−1^), specifically *Streptococcus mutans* and *S. sobrinus*, which are clinically relevant pathogens associated with oral and dental infections. The superior antimicrobial efficacy observed for Ag–ZnO NCs was likely due to enhanced electrostatic interactions between the negatively charged bacterial cell surfaces and the Ag ions present in the nanocomposites. The research also documented that the most differentiating nanoproduct was Ag–ZnO NCs. At the same time, ion release and crystallite size exhibited a major discriminating influence on the observed differences among the samples. The adsorption kinetic models revealed that Ag ions were released more rapidly and efficiently from Ag–ZnO NCs compared to Cu ions and Cu–ZnO NCs. The use of ATR–FTIR supported by FAMEs and SEM analytical techniques demonstrated their strong potential for molecular-level cell analysis, complementing biochemical and microbiological studies to enable a comprehensive understanding of MONC–bacterial cell interactions. These interactions induced alterations in protein secondary structures as well as modifications in lipid composition, including changes in saturated fatty acid content. SEM micrographs revealed that the synthesized MONCs disrupted the surface integrity of both Gram-positive bacteria, causing visible membrane damage, perforations, and subsequent leakage of intracellular contents. Moreover, treatment with Ag–ZnO NCs impaired chain and cluster formation in both bacterial strains, whereas this effect following Cu–ZnO NCs treatment was observed only in *S. sobrinus* cultures.

Overall, these findings demonstrate that the newly developed nanocomposites exhibit strong antimicrobial activity, highlighting Ag–ZnO and Cu–ZnO NCs as promising next-generation materials for the inactivation of oral pathogenic microorganisms. Future studies should focus on optimizing dosage and concentration thresholds to ensure therapeutic efficacy while maintaining biocompatibility for safe biomedical applications.

## Figures and Tables

**Table 1:** Basic characteristics and selective culture media of reference microorganisms from ATCC.

Microorganisms (ATCC)	Gram Nature	Media Reference Number	Formula [g L^−1^]
*S. mutans* 25175	positive	BD 237500	(37 g) Calf Brains Infusion, Beef Heart Infusion, Proteose Peptone, Dextrose, Sodium Chloride, Disodium Phosphate
*S. sobrinus* 33478			

**Table 2:** Selected typical FTIR spectra bands of chemical compounds for tested cells, untreated and treated with MONCs [84,87].

Position (cm^−1^) of Bands	Main Contribution
1240	Distribution of phosphate-containing compounds: phospholipids, nucleic acids, phosphorylated carbohydrates. Variations in the phosphorylation degree of carbohydrates and glycoproteins.
1545/1658	Protein structural modifications.
2924/1658	Relative lipid-to-protein ratio.
2924/2955	Structural modifications in saturated lipids, including chain length and branching degree.

**Table 3:** Elemental composition of Ag–ZnO and Cu–ZnO nanocomposites determined by EDS, expressed as weight percentages (n = 3; ±SD).

Ag–ZnO NCs		Cu–ZnO NCs	
Element	Weight, % (±SD)	Element	Weight, % (±SD)
O	22.49 ± 5.54	O	22.56 ± 0.79
Zn	63.18 ± 1.85	Zn	67.88 ± 2.11
Ag	14.12 ± 2.72	Cu	9.55 ± 1.84

**Table 4:** The ζ-potential, hydrodynamic diameter, and pH values of the MONCs and the reference ZnO nanoparticles were measured in aqueous (Millipore H_2_O) suspension.

MONCs/NPs	ζ (mV) *	DLS (nm) **	pH
Ag–ZnO	−22	1296	6.5
Cu–ZnO	−2.6	1769	6.5
ZnO	12	2059	6.5

SD: * ±0.8 mV; ** ±400 nm.

## Data Availability

The original contributions presented in this study are included in the article/Supplementary Material. Further inquiries can be directed to the corresponding author.

## Supplementary Materials

The following supporting information can be downloaded at: https://www.mdpi.com/article/10.3390/ma19081634/s1, Figure S1: Box-and-whisker plots of phosphate-related biochemical parameters in S. *mutans* exposed to Ag–ZnO and Cu–ZnO nanocomposites at the minimum inhibitory concentration (MIC) compared with untreated controls (A–C). Data are presented as medians with interquartile ranges and minimum and maximum values from vector-normalized infrared spectra. Statistical significance was assessed using the Mann–Whitney test, with * indicating *p* < 0.05; Figure S2: Box-and-whisker plots of phosphate-related biochemical parameters in S. *sobrinus* exposed to Ag–ZnO and Cu–ZnO nanocomposites at the minimum inhibitory concentration (MIC) compared with untreated controls (A–C). Data are presented as medians with interquartile ranges and minimum and maximum values from vector-normalized infrared spectra. Statistical significance was assessed using the Mann–Whitney test, with * indicating *p* < 0.05; Table S1: The percentages of fatty acids in the FAME profiles of S. *mutans* treated with MONMs/NPs and in the control; Table S2: The percentages of fatty acids in the FAME profiles of S. *sobrinus* treated with MONMs/NPs and in the control.

## Abbreviations

The following abbreviations are used in this manuscript:

NCs	nanocomposites
MONCs	metal oxide nanocomposites
TEM	transmission electron microscopy
XRD	X-ray diffraction
DLS	dynamic light scattering
PFO	pseudo-first-order (PFO) expression
PSO	pseudo-second-order (PSO) expression
MIC	minimum inhibitory concentration
ATR–FTIR	Attenuated Total Reflectance Fourier Transform Infrared
SE	scanning electron microscopy
FAMEs	fatty acid methyl esters
AMR	antimicrobial resistance
CDC	Centers for Disease Control and Prevention
MRSA	methicillin-resistant Staphylococcus aureus
VRE	vancomycin-resistant enterococci
DNA	deoxyribonucleic acid
ATP	adenosine triphosphate
BPEI	branched polyethylenimine
PVP	polyvinylpyrrolidone
NPs	nanoparticles
STEM	scanning transmission electron microscopy
HAADF	high-angle annular dark field
EDS	energy-dispersive X-ray spectroscopy
COD	Crystallography Open Database
ATCC	American Type Culture Collection
SD	standard deviation
ANOVA	one-way analysis of variance
HSD	honestly significant difference
PCA	principal component analysis
FL-AAS	flame atomic absorption spectroscopy
NMs	nanomaterials
ROS	reactive oxygen species
^1^O_2_	singlet oxygen
O_2_·^−^	superoxide anion
·OH	hydroxyl radical
OH^−^	hydroxyl ion
LB	Luria–Bertani medium
CMA	L-glutamine
CMB	Ultraglutamine
EPS	extracellular polymeric substances
