# Supplementary material for: Metal Oxide Nanocomposites as Next-Generation Antimicrobial Agents Against Oral Cariogenic Pathogens: Mechanistic Actions of Ag–ZnO and Cu–ZnO on S. mutans and S. sobrinus
Source: Materials (Basel). 2026 Apr 19;19(8):1634. doi: 10.3390/ma19081634 (PMC13118030; doi:10.3390/ma19081634)
Supplement: Supplementary file 1 [file materials-19-01634-s001.zip › Supplementary materials.pdf]

Supplementary materials

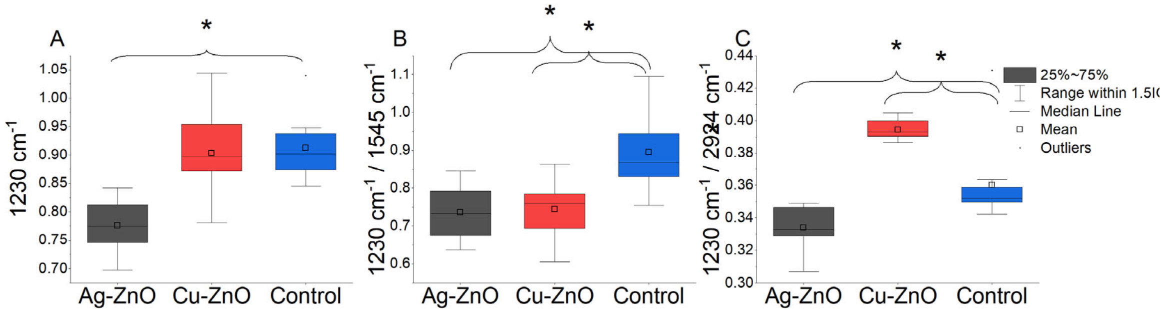

Figure. S1. Box-and-whisker plots of phosphate-related biochemical parameters in *S. mutans* exposed to Ag–ZnO and Cu–ZnO nanocomposites at the minimum inhibitory concentration (MIC) compared with untreated controls (A–C). Data are presented as medians with interquartile ranges and minimum and maximum values from vector-normalized infrared spectra. Statistical significance was assessed using the Mann–Whitney test, with * indicating $p < 0.05$.

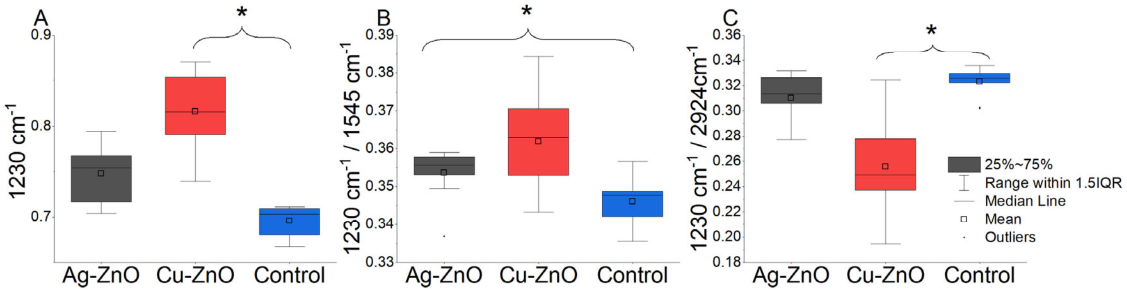

Figure. S2. Box-and-whisker plots of phosphate-related biochemical parameters in *S. sobrinus* exposed to Ag–ZnO and Cu–ZnO nanocomposites at the minimum inhibitory concentration (MIC) compared with untreated controls (A–C). Data are presented as medians with interquartile ranges and minimum and maximum values from vector-normalized infrared spectra. Statistical significance was assessed using the Mann–Whitney test, with * indicating $p < 0.05$.

Table S1. The percentages of fatty acids in the FAME profiles of *S. mutans* treated with MONMs/NPs and in the control.

Fatty acid	Control	Ag-ZnO	Cu-ZnO	ZnO
		%		
14:0	2.36	2.91	2.61	2.74
16:0	30.21	31.52	30.99	25.50
18:0	6.74	8.06	6.43	3.82
16:1 ω5*c*	0.74	0.21	0.53	1.08
16:1 ω9*c*	4.06	3.53	3.17	7.32
16:1 ω7*c*/ 16:1 ω6*c*	0.89	1.67	1.87	1.52
18:1 ω7*c*	27.59	22.30	17.29	30.25
18:1 ω9*c*	7.88	14.04	26.00	9.94
19:1 *iso* I	1.05	0.42	0.18	1.95
20:1 ω7*c*	1.22	0.65	0.78	0.90
20:1 ω9*c*	17.25	14.69	10.16	14.98

Table S2. The percentages of fatty acids in the FAME profiles of *S. sobrinus* treated with MONMs/NPs and in the control.

Fatty acid	Control	Ag-ZnO	Cu-ZnO	ZnO
		%		
11:0 *iso* 3OH	0.00	0.00	2.66	0.00
14:0	5.04	5.21	5.24	4.93
15:0 *iso*	4.10	2.76	2.46	2.72
15:0 *anteiso*	21.69	16.77	15.72	18.10
16:0	20.15	23.58	23.57	22.85
17:0 *iso*	3.30	1.52	1.07	1.62
17:0 *anteiso*	8.85	4.77	3.42	4.92
18:0	5.86	7.21	4.92	7.33
19:0 *iso*	3.47	2.07	1.12	2.13
19:0 *anteiso*	5.27	3.44	1.81	3.57
20:0	2.59	2.98	1.32	2.05
16:1 ω9*c*	1.39	1.48	1.24	1.50
16:1 ω7*c*/ 16:1 ω6*c*	0.60	1.41	2.06	1.37
18:1 ω7*c*	9.41	10.65	7.41	10.66
18:1 ω9*c*	4.30	11.11	23.30	11.33
20:1 ω9*c*	4.00	5.06	2.67	4.91
